# A growing concern: How soon will India run out of water?

**Published:** 2011-12

**Authors:** Rajmohan Panda

**Affiliations:** Public Health Foundation of India, New Delhi, India

Water resources of a country constitute one of its vital assets. India receives an average of 4000 billion cubic meters of rainfall every year, confined to the monsoon season (June through September). Unfortunately, due to lack of storage and crumbling infrastructure, only 18% can be used (1). Apart from the water available in the various rivers of the country, groundwater forms an important source of water for drinking, irrigation, industrial and other uses. Groundwater is a vital resource, with a large fraction of the population relying on the resource directly or indirectly for livelihoods. Groundwater accounts for about 50-80% of domestic water use and 45-50% of irrigation in the country (2). This heavy reliance on groundwater for both domestic water and irrigation purposes is now approaching its limit as an increasing number of aquifers reach unsustainable levels after decades of exploitation. Overall, India has around 432 cubic kilometres of annual replenishable groundwater resources. With a net annual groundwater availability of 399 cubic kilometres, the net withdrawals in 2004 amounted to 58% of the net annually available resource (3). However there are several regional and intra-state variations. According to the 2004 nationwide assessment, 29% of the groundwater blocks were in the semi-critical, critical, or overexploited categories (3).

In already large and rapidly growing segments of the economy and in many of India’s most productive regions, unlimited groundwater use is no longer sustainable. A crisis situation now exists in a number of states for example In Punjab, groundwater in 75% of blocks (sub-district administration units) is overdrawn; the corresponding fraction in Rajasthan is 60% (3). The situation is deteriorating at a rapid pace. The gravity of the situation can be appreciated from the fact that the proportion of overexploited blocks nationwide has tripled from 5% to 15% between 1995 and 2004 (3). The World Bank estimate in 2005 warned that if the current trends continue, 60% of all aquifers in India will be in a critical condition within 20 years (4). In a recent study, Rodell et al. used Gravity Recovery and Climate Experiment (GRACE) satellites operated by NASA and the German Aerospace Center (DLR) to calculate the loss rate to be around 20% higher than the Indian authorities have previously estimated (5).

The potential social and economic consequences of weak or nonexistent groundwater management are serious because aquifer depletion is concentrated in many of the most populated and economically productive areas in the country. Given water’s cross-cutting linkages, the implications are disturbing for the attainment of the Millennium Development Goals, for sustaining economic growth and local livelihoods, and for environmental and fiscal sustainability. Falling water tables would also be likely to affect progress in education, health, gender, child mortality, poverty, and hunger.

The exploitation of groundwater resources should be regulated so as not to exceed the recharging possibilities, as well as to ensure social equity. The detrimental consequences of over-exploitation of groundwater on the environment need urgent attention and co-operation between the central (federal) and State Governments. The Constitution lists “water supplies” under the State List while the Central Government is in “overall planning for the development of groundwater resources”. Management of groundwater thus suffers from fragmentation of responsibility at both central and state levels.

**Figure:**
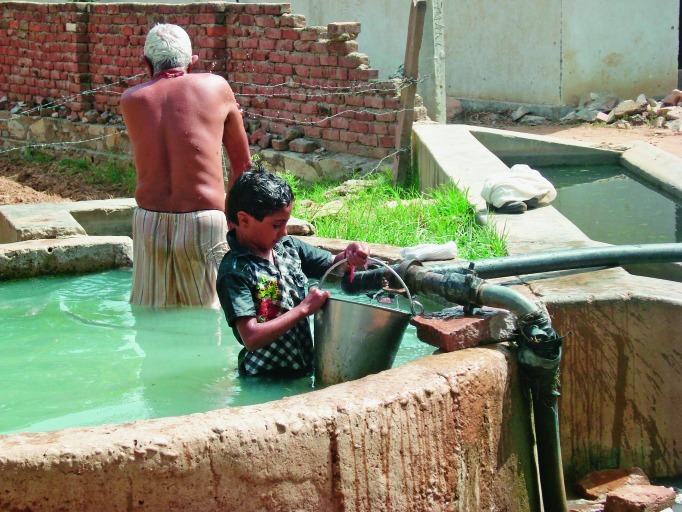
Photo: Courtesy of Dr Raj Panda, personal collection

The tragedy of India’s water scarcity is that the crisis could have been largely avoided with better water management practices. There has been a distinct lack of attention to water legislation, water conservation, efficiency in water use, water recycling, and infrastructure. The National Groundwater Recharge Master Plan, which provides a nationwide assessment of the groundwater recharge potential, estimates that through dedicated artificial recharge structures in rural areas and rooftop water harvesting structures in urban areas a total of 36 billion cubic meters can be added to groundwater recharge, at a cost of approximately US$ 6 billion (€ 4.4) (6). Artificial groundwater recharge can only be a part of the solution in certain settings, but is not a holistic approach for sustainable development and management that is needed for addressing the problem of overexploited aquifers storage. Thus, efforts to address excessive groundwater exploitation must also concentrate largely on the promotion of appropriate measures to manage demand, too. Dry-season crop planning, adoption of modern precision irrigation technologies, restrictions to control groundwater abstraction either voluntary (through community based management approaches) or through regulatory measures should be part of the broader solution. Groundwater management interventions should also be structured to serve the basic interests of the users, taking into account the socioeconomic realities of each particular groundwater setting.

**Figure:**
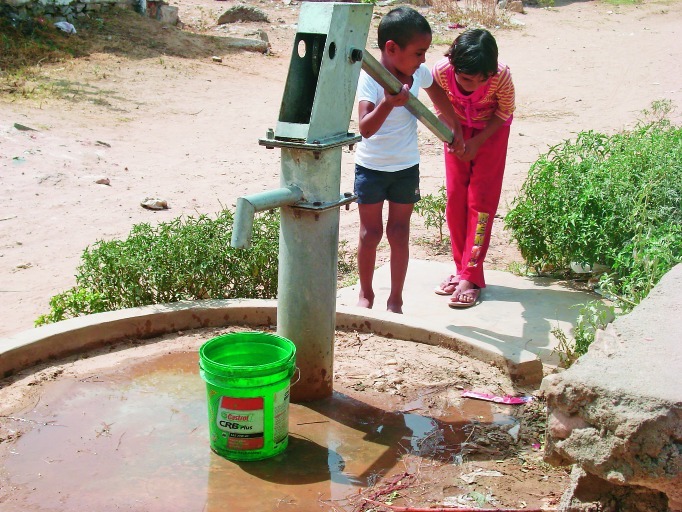
Photo: Courtesy of Dr Akshaya Srivastav, personal collection
